# Earliest trace, so far, of community genetics as a distinct concept

**DOI:** 10.1007/s12687-012-0083-3

**Published:** 2012-02-12

**Authors:** Leo P. ten Kate

**Affiliations:** VU University Medical Center, Amsterdam, The Netherlands

According to Modell and Kuliev (1998), the history of community genetics as a distinct concept in medicine started in 1981 with WHO in Geneva. This was about the same time that community genetics was introduced in biology for research on interacting populations in a shared environment (Ten Kate et al. 2010). We do not know whether either or both of these early uses can be traced back to even earlier usage.

More recently, I found one earlier mention of community genetics in PubMed. The authors of that 1975 report describe the results of cytogenetic analysis in 136 patients referred to a genetic service, located at the Children’s Medical Center, Tulsa, Oklahoma, and conclude that the results of their genetic clinic ‘demonstrate its need and value to the community’ (Coldwell et al. 1975). The clinic was the initiative of the first author, James Coldwell, a pediatrician, who informed me that he first was involved in screening children for phenylketonuria in Oklahoma, and subsequently spent some time with Victor McKusick at John Hopkins, before developing his genetic service in Tulsa. He does not think that he borrowed the term community genetics from someone else. It suggests that the idea of having a genetic clinic for the benefit of the community of itself leads to the concept of community genetics.

Alternatively, the parallel of community genetics with community medicine catches the eye. The oldest reference to community medicine in PubMed dates from the year 1920, and there are 63 references to papers with community medicine in their title in the 1960s and 248 in the 1970s. Viewed from this perspective, one may even start to wonder why it took so long before someone introduced the term community genetics.
